# Operationalizing AI-Enabled Cardiovascular Biomarkers: A Clinician-Centered Framework for Validation, Governance, and Workflow Integration

**DOI:** 10.2196/103401

**Published:** 2026-08-28

**Authors:** Dabeluchi Chiedozie Ngwu

**Affiliations:** 1 Department of Surgery, King Abdullah Hospital, Al Nakhil, Bisha, Asir, 67714, Saudi Arabia, 966 543423453

**Keywords:** artificial intelligence, cardiovascular biomarkers, digital biomarker, clinical validation, clinical decision support systems, workflow integration, governance

## Abstract

Cardiovascular biomarkers are increasingly extracted or interpreted using artificial intelligence (AI) applied to electrocardiograms, imaging, laboratory measurements, electronic health records, wearable devices, and longitudinal data. Predictive performance alone, however, does not establish that a measurement is valid, that a model is transportable and calibrated, or that acting on its output improves care. Existing resources provide essential but complementary foundations: the US Food and Drug Administration–National Institutes of Health (FDA-NIH) BEST (Biomarkers, EndpointS, and other Tools) resource standardizes biomarker terminology; the V3 and V3+ frameworks address verification, analytical validation, clinical validation, and usability of digitally measured signals; prediction-model and trustworthy-AI guidance addresses reporting, risk of bias, early clinical evaluation, and deployability; and regulatory qualification pathways evaluate evidence within a defined context of use. A remaining practical challenge is translating these complementary requirements into accountable clinical action within cardiovascular workflows. This viewpoint proposes a clinician-centered operational framework organized around validation, governance, and workflow integration. For each pillar, it identifies accountable actors, required steps, documented outputs, and escalation or stop rules. Validation establishes whether the input measurement and model are fit for the intended population and decision. Governance assigns institutional authorization, clinical ownership, version control, monitoring, and authority to restrict, pause, or withdraw the intervention. Workflow integration specifies who receives the output, what confirmatory action follows, how disagreement is handled, and how decisions are documented. A worked example of an AI-enabled electrocardiographic screening output for possible left ventricular systolic dysfunction illustrates the pathway from local evaluation to echocardiographic confirmation and lifecycle monitoring. The framework does not replace established validation or regulatory standards; it operationalizes them as a clinician-centered and institutionally accountable pathway from validated signal to governed, patient-facing action.

## Introduction

Cardiovascular biomarkers influence consequential decisions, including diagnosis, triage, preventive treatment, referral, device therapy, and longitudinal monitoring. Artificial intelligence (AI) is expanding this landscape by extracting or interpreting signals from electrocardiograms (ECGs), imaging, laboratory measurements, electronic health records (EHRs), wearable devices, omics platforms, and longitudinal clinical data. These methods may reveal patterns that conventional thresholds or models do not capture and support earlier recognition or more individualized assessment [1-12]. Their clinical value, however, depends on more than performance in a development dataset.

Terminology is important because several related objects are often conflated. Following the US Food and Drug Administration–National Institutes of Health (FDA-NIH) BEST (Biomarkers, EndpointS, and other Tools) resource, a biomarker is a defined characteristic measured as an indicator of normal biological processes, pathogenic processes, or responses to an exposure or intervention [13]. A digital biomarker is a biomarker measured or derived using digital technology [14,15]. In this viewpoint, an AI-enabled cardiovascular biomarker is a cardiovascular biomarker whose extraction, quantification, combination, or interpretation materially depends on an AI method. Digital biomarkers and AI-enabled biomarkers overlap, but neither wholly contains the other: a digitally captured heart-rate signal may require no AI, whereas AI may interpret conventional laboratory or imaging measurements. A prediction or risk score is a model-generated estimate and is not automatically a biomarker. A complete clinical decision-support intervention comprises the data input, model or interpretive process, output, interface, intended user, response pathway, and oversight arrangements.

These distinctions matter because an AI-enabled biomarker output may be associated with disease without being suitable for clinical action. A model may discriminate well yet be poorly calibrated, lose performance across settings or patient groups, or trigger testing and treatment whose benefits and harms have not been established. Reliability concerns the consistency or reproducibility of a measurement, whereas validity concerns whether it accurately captures the intended characteristic. External validation tests performance in data separated from development by place, time, population, or setting. Calibration concerns agreement between predicted probabilities and observed event frequencies in the intended population, particularly around thresholds used for action. Clinical utility asks whether using the output improves decisions or patient-relevant outcomes enough to justify its consequences [2-4,16-23]. Predictive performance in a development dataset is therefore only one component of validation and does not establish measurement validity, transportability, calibration, or clinical utility in the intended setting.

Existing frameworks provide complementary foundations. BEST standardizes biomarker terminology and categories [13]. V3 addresses verification, analytical validation, and clinical validation for biometric monitoring technologies, while V3+ extends this foundation to usability validation for sensor-based digital health technologies [14,15]. Cardiovascular guidance addresses the predictive and clinical utility of novel biomarkers and models [2-4]. TRIPOD+AI (Transparent Reporting of a Multivariable Prediction Model for Individual Prognosis or Diagnosis + Artificial Intelligence) and PROBAST+AI (Prediction Model Risk of Bias Assessment Tool + Artificial Intelligence) address reporting, risk of bias, and applicability; DECIDE-AI (Developmental and Exploratory Clinical Investigations of Decision Support Systems Driven by Artificial Intelligence) addresses early-stage clinical evaluation; and FUTURE-AI (Fairness, Universality, Traceability, Usability, Robustness, and Explainability in Artificial Intelligence) addresses trustworthy and deployable AI [23-26]. FDA and European Medicines Agency qualification pathways also anchor evidence to a defined context of use in medical product development [27,28]. V3+ focuses on usability, whereas the workflow-integration pillar considered here addresses the broader clinical pathway through which an output is received, confirmed, acted upon, documented, and monitored.

This viewpoint does not propose a new biomarker taxonomy or replace established validation standards. It integrates these complementary requirements into a clinician-centered implementation pathway that links fit-for-purpose evidence to named clinical and institutional responsibilities, predefined clinical responses, and lifecycle monitoring. The framework is organized around 3 interconnected pillars: validation, governance, and workflow integration. A visual abstract of the proposed framework is presented in Multimedia Appendix 1.

## From Biomarker Signal to Clinical Intervention

Biomarker translation can fail before AI is introduced. The underlying assay, sensor, image acquisition process, or data stream may be unstable; the development cohort may be narrow; the outcome may be poorly defined; or the proposed use may not correspond to a meaningful clinical decision. Sample handling, device characteristics, preprocessing, timing, biological variability, kidney function, comorbidity, medication use, and missing data can all alter the behavior of a cardiovascular signal. If the input is unreliable or invalid, a more sophisticated model cannot repair the evidentiary foundation [2,3,16-19].

Transportability presents a second challenge. Cardiovascular populations and workflows vary across hospitals, laboratories, ECG systems, imaging platforms, EHRs, referral practices, and treatment pathways. Average performance may conceal important failures across age, sex, comorbidity, ancestry or ethnicity (when these data are appropriately collected), kidney function, device access, and care setting. Because local prevalence also changes positive and negative predictive values, a threshold developed in a tertiary referral cohort may generate an unacceptable false-positive burden in primary care. External and local validation must therefore examine calibration, subgroup performance, and the consequences of the proposed threshold, not discrimination alone [23,24,29-31].

Clinical utility remains distinct from statistical performance. A reproducible and externally validated output may add little beyond existing assessment, identify patients for whom no beneficial response is available, or generate unnecessary testing, anxiety, treatment, and cost. Incremental performance, decision-curve analysis, pragmatic evaluation, and patient-relevant outcomes can help determine whether acting on the output is preferable to current care [4,17,20,22,32]. The relevant unit of evaluation is therefore the complete clinical decision-support intervention: who receives the output, what action follows, and what happens to the patient.

AI can nevertheless add meaningful capabilities by combining multimodal signals, identifying nonlinear relationships, supporting phenotype discovery, and enabling longitudinal interpretation. It may also support lifecycle monitoring by detecting calibration drift, changes in data quality, and subgroup deterioration. These capabilities become clinically meaningful only when the output is tied to a defined intended use, accountable ownership, and an evaluable response pathway. Otherwise, AI may scale weak evidence or ambiguous action more rapidly than conventional biomarker development [5,8-12,21,25,26,32-34].

## A Clinician-Centered Operational Framework

### Overview

The 3 pillars are connected, rather than parallel, checklists. Validation determines what the output can legitimately support. Governance determines who may authorize, monitor, modify, pause, or withdraw it. Workflow integration determines what happens when the output enters clinical care. Each pillar is therefore defined by accountable actors, required steps, documented outputs, and escalation or stop rules.

### Validation

Accountable actors include the developer or vendor, a local clinical lead, an expert in the relevant measurement domain, and a biostatistics or data-science team. Their first task is to define the intended use: the target population, setting, decision, timing, comparator, and actions that may follow. Verification and analytical validation should establish that the acquisition system, assay, device, preprocessing pipeline, and data transfer perform as intended. Clinical validation should establish the relationship between the measured characteristic or model output and the relevant clinical state or outcome [13-15].

Where an AI model produces a probability, classification, or risk score, evaluation should include separation from development data, discrimination, calibration, clinically relevant subgroup performance, robustness to local devices and data pipelines, and positive or negative predictive value in the intended population. Thresholds should be evaluated in relation to downstream action rather than selected for statistical convenience. Clinical utility should determine whether the output adds value beyond current assessment and whether expected benefits justify confirmatory testing, referral, treatment, or monitoring [4,20,22-24,26,27,32]. When important subgroup differences are identified, evaluation should move beyond reporting them to determining whether data revision, threshold adjustment, workflow safeguards, restricted use, or nondeployment is required.

The resulting local evidence report should specify the approved population, exclusions, model version, data requirements, threshold, uncertainty, expected downstream burden, and residual limitations. Deployment should not proceed when the input is insufficiently reliable or valid, performance is not transportable or calibrated for the intended setting, important subgroup risks remain unresolved, or no justifiable clinical response follows.

### Governance

Governance requires both a named clinical service owner and a multidisciplinary institutional oversight function. The exact structure may vary, but it should include clinical, informatics, data-science, quality and safety, privacy, and legal or risk-management expertise, with patient or public perspectives incorporated when warranted by the intended use and patient-facing consequences. Vendors may provide technical documentation and model updates, but the institution retains responsibility for local authorization and oversight and should maintain an independent record of the deployed version.

Before deployment, the oversight body should approve the intended use, eligible population, prohibited uses, data dependencies, clinical owner, monitoring metrics, review cadence, incident-reporting route, and procedure for material updates. Version changes, altered input pipelines, new devices, population shifts, or workflow redesign may invalidate prior assumptions and should trigger proportionate reassessment. Prespecified monitoring criteria may include material calibration deterioration, unacceptable subgroup disparities, increased false-positive burden, clinically important missed cases, harmful downstream action, or substantial changes in data quality or software behavior [25,26,32-37].

Governance should produce formal authorization, a named accountable owner, a version and change log, a monitoring protocol, and procedures for recalibration, restricted use, temporary suspension, and withdrawal. It is effective only when someone has both the information and authority to act when performance or clinical consequences become unacceptable.

### Workflow Integration

Workflow integration is owned by the clinical pathway rather than the algorithm. The clinical service lead, intended users, informatics team, and relevant nursing or allied professionals should determine who receives the output, when and where it appears, what confirmatory action is appropriate, how urgency is communicated, and how uncertainty or disagreement is documented. The output should be designed around a decision point rather than added as an unprioritized dashboard item or interruptive alert.

A defined response pathway specifies the recipient; the threshold or conditions that trigger review; permissible actions; any required confirmation before treatment; escalation and de-escalation rules; when an override rationale is required; and how responsibility transfers across teams. Documentation should be proportionate to clinical risk. When an AI output conflicts with clinical judgment, the pathway should preserve clinician authority while making high-risk disagreements visible for review. Patient communication should be specified when the result leads to testing, referral, or treatment discussion.

**Figure 1.**
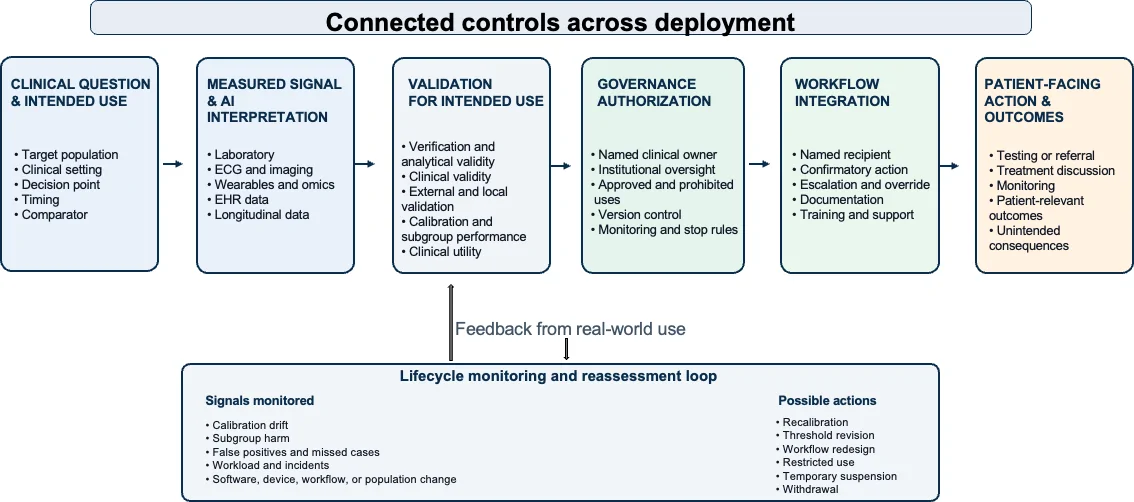
Clinician-centered operational pathway for AI-enabled cardiovascular biomarkers. AI: artificial intelligence; ECG: electrocardiogram; EHR: electronic health record.

The pathway should be written, tested, and embedded in the relevant clinical system. Implementation readiness also requires role-specific training so that intended users understand the output, its limitations, the required confirmatory actions, and the circumstances in which escalation or override is appropriate. Deployment should be delayed when the recipient is unclear, confirmatory capacity is unavailable, the alert cannot be acted on within the required time, the output encourages unsupported treatment, or responsibility is fragmented across teams. Workflow simulation, silent or shadow-mode testing, and early prospective evaluation can identify these failures before broad implementation [9-12,26,32]. Figure 1 summarizes the clinician-centered operational pathway from intended use and signal measurement through validation, governance authorization, workflow integration, patient-facing action, and lifecycle reassessment.

The pathway begins with a defined clinical question and intended use, followed by measurement and AI-enabled interpretation, validation for the intended population and decision, governance authorization, workflow integration, and patient-facing action. Validation, governance, and workflow integration operate as connected controls across deployment. Postdeployment monitoring evaluates calibration drift, subgroup harm, false-positive and missed-case patterns, workload, incidents, and changes in software, devices, workflows, or populations. Findings may trigger recalibration, threshold revision, workflow redesign, restricted use, temporary suspension, or withdrawal. The pathway, therefore, does not end at model validation; authorization, clinical response, and lifecycle reassessment determine whether an output becomes a safe clinical decision-support intervention.

## Applying the Framework: AI-ECG Screening for Left Ventricular Systolic Dysfunction

Consider an AI-enabled ECG model intended to identify patients with possible left ventricular systolic dysfunction who may benefit from echocardiography. Development studies have shown that AI can detect a signal associated with low ejection fraction from a standard ECG, and pragmatic evaluation has shown that presenting this output in routine primary care can increase detection of low ejection fraction [38,39]. Its intended use must remain narrow: the output is a screening prompt for confirmatory assessment, not a diagnosis of heart failure or an independent indication for treatment.

Validation would require confirmation that the locked model is compatible with local ECG acquisition, preprocessing, and data-transfer systems, followed by testing in a local or otherwise suitably representative population independent of model development. Evaluation should include discrimination, calibration, positive predictive value, subgroup performance, and the expected number of echocardiograms generated per confirmed case. A threshold acceptable in a referral population may produce excessive false positives or overwhelm echocardiography capacity in lower-prevalence primary care.

Governance would require institutional authorization, a named clinical owner, and documented responsibility for version control and monitoring. Relevant measures would include false-positive and false-negative patterns, subgroup performance, echocardiography yield, time to confirmation, alert burden, and inappropriate downstream action. Material performance deterioration or a change in the software or data pipeline should trigger reassessment and, when necessary, restricted use or suspension.

Within the workflow, the output would reach a named ordering clinician or designated results-management team with a clear recommendation for clinical review and, where appropriate, confirmatory echocardiography. The pathway would specify urgency, referral options, documentation, and how to respond when recent imaging or an alternative explanation is already available. No diagnosis of heart failure or disease-specific treatment should follow solely from the AI-ECG output. The example illustrates how a technically valid prediction becomes part of a safer clinical decision-support intervention only when evidence, ownership, confirmation, and monitoring are connected.

## Operational Requirements

Table 1 translates the 3 pillars and the cross-cutting lifecycle-monitoring function into minimum operational requirements. The requirements are role-based rather than tied to a single committee structure because institutions differ in size and resources. Across settings, explicit ownership, documented evidence, a tested response pathway, and authority to restrict or stop use should remain nonnegotiable.

**Table 1.** Minimum operational requirements for validation, governance, workflow integration, and lifecycle monitoring of AI-enabled cardiovascular biomarkers.

Domain	Accountable actors	Minimum operational requirements	Required outputs and action threshold
Validation	Developer or vendor; local clinical lead; measurement-domain expert; biostatistics or data-science team	Define intended use and population; verify acquisition and preprocessing; conduct analytical and clinical validation; assess transportability, calibration, subgroup performance, and clinical utility	Local evidence report specifying population, exclusions, model version, decision threshold, uncertainty, and limitations. Do not deploy when validity, calibration, transportability, subgroup safety, or actionable utility is inadequate.
Governance	Named clinical service owner; multidisciplinary institutional oversight body; quality, safety, and informatics representatives	Authorize intended and prohibited uses; document data dependencies and version; assign monitoring; review incidents and material updates; define restriction, pause, and withdrawal procedures	Authorization record, accountable owner, change log, and monitoring plan. Reassess, restrict, pause, or withdraw use after material performance deterioration, inequity, harmful consequences, or major system change.
Workflow integration	Clinical pathway owner; intended clinicians; informatics; nursing and allied professionals as relevant	Identify recipient, timing, interface, confirmatory action, urgency, escalation, override, documentation, patient communication, and training requirements	Written and tested response pathway, workflow map, and training materials. Delay deployment when responsibility is unclear, action capacity is unavailable, alert burden is unsafe, or unsupported treatment may result.
Lifecycle monitoring (cross-cutting)^a^	Clinical owner; governance body; analytics or quality team; vendor as applicable	Track calibration, yield, data quality, subgroup performance, missed cases, false positives, downstream actions, workload, incidents, and version changes	Monitoring report and corrective-action record. Trigger recalibration, threshold revision, workflow redesign, restricted use, suspension, or withdrawal according to prespecified risk-based criteria.

a Lifecycle monitoring is cross-cutting and supports all 3 pillars.

## Implications for Evaluation and Reporting

Investigators, peer reviewers, health systems, and regulators should evaluate AI-enabled cardiovascular biomarker studies beyond discrimination alone. Reports should clarify whether the object under evaluation is a biomarker, digital biomarker, prediction model, or complete clinical decision-support intervention; define the intended use and target population; describe measurement verification and validity; report external validation, calibration, and clinically relevant subgroup performance; and explain the comparator and downstream consequences. When deployment is proposed, reports should also identify the accountable owner, response pathway, prohibited uses, monitoring plan, and criteria for recalibration, suspension, or withdrawal.

The evidentiary question, therefore, shifts from “Does the model predict?” to “Are the measured signal, model, institution, and response pathway jointly fit for the intended use?” A valid measurement and a well-performing model do not by themselves establish the clinical value or safety of deployment, and health systems should not infer either from regulatory status, technical performance, or publication. Prospective evaluation should examine patient-relevant outcomes, unintended consequences, clinician workload, equity, and the behavior of the complete clinical decision-support intervention over time [25,26,32].

## Conclusion

AI can expand the information obtainable from cardiovascular biomarkers and related clinical data, but technical performance alone does not establish that an input is valid, an output is transportable and calibrated, or that acting on it improves care.

The proposed framework links established evidentiary standards to clinical implementation by connecting validation, accountable governance, defined response pathways, and lifecycle monitoring. The worked AI-ECG example shows why these functions must be designed together. Clinical trust should therefore be earned at the level of the complete clinical decision-support intervention: a validated signal, a fit-for-purpose model, accountable clinical and institutional ownership, a defined response pathway, and continuing evidence that the intervention remains safe and useful in practice.

## Supplementary material

10.2196/103401 Multimedia Appendix 1 Visual abstract.

## Abbreviations

AI: artificial intelligence
BEST: Biomarkers, EndpointS, and other Tools
DECIDE-AI: Developmental and Exploratory Clinical Investigations of Decision Support Systems Driven by Artificial Intelligence
ECG: electrocardiogram
EHR: electronic health record
FDA: US Food and Drug Administration
FUTURE-AI: Fairness, Universality, Traceability, Usability, Robustness, and Explainability in Artificial Intelligence
NIH: National Institutes of Health
PROBAST+AI: Prediction Model Risk of Bias Assessment Tool + Artificial Intelligence
TRIPOD+AI: Transparent Reporting of a Multivariable Prediction Model for Individual Prognosis or Diagnosis + Artificial Intelligence
